# Cell-based cytotoxicity assays for engineered nanomaterials safety screening: exposure of adipose derived stromal cells to titanium dioxide nanoparticles

**DOI:** 10.1186/s12951-017-0285-2

**Published:** 2017-07-11

**Authors:** Yan Xu, M. Hadjiargyrou, Miriam Rafailovich, Tatsiana Mironava

**Affiliations:** 1 Department of Materials Science and Engineering, Stony Brook University, Stony Brook, NY USA; 2 Department of Life Sciences, New York Institute of Technology, Old Westbury, NY USA

**Keywords:** Adipose derived stromal cells, Titanium dioxide, Cytotoxicity assays, Nanomaterials safety screening

## Abstract

**Background:**

Increasing production of nanomaterials requires fast and proper assessment of its potential toxicity. Therefore, there is a need to develop new assays that can be performed in vitro, be cost effective, and allow faster screening of engineered nanomaterials (ENMs).

**Results:**

Herein, we report that titanium dioxide (TiO_2_) nanoparticles (NPs) can induce damage to adipose derived stromal cells (ADSCs) at concentrations which are rated as safe by standard assays such as measuring proliferation, reactive oxygen species (ROS), and lactate dehydrogenase (LDH) levels. Specifically, we demonstrated that low concentrations of TiO_2_ NPs, at which cellular LDH, ROS, or proliferation profiles were not affected, induced changes in the ADSCs secretory function and differentiation capability. These two functions are essential for ADSCs in wound healing, energy expenditure, and metabolism with serious health implications in vivo.

**Conclusions:**

We demonstrated that cytotoxicity assays based on specialized cell functions exhibit greater sensitivity and reveal damage induced by ENMs that was not otherwise detected by traditional ROS, LDH, and proliferation assays. For proper toxicological assessment of ENMs standard ROS, LDH, and proliferation assays should be combined with assays that investigate cellular functions relevant to the specific cell type.

## Background

The growing annual production of engineered nanomaterials (ENMs) has led to a proportional increase in the chance of occupational and consumer exposure and has raised serious concerns about their environmental, health and safety impact. To be able to properly screen and predict the potential toxicity of ENMs, sensitive and reliable in vitro assays need to be developed as soon as possible [1–3]. Ideally, such assays should be relevant to a real life exposure scenario, be cost effective to allow for massive ENMs screening, and measure common modes of cellular responses [4]. An approach like this will provide researchers with consistent and accurate tools for comparing cellular responses to various ENMs, identify properties of materials causing the response and provide insights into the mechanism of toxicity [2, 5].

However, before developing such methods we need to have a clear strategy as to how to address ENMs risk in relevant cellular models. There are several different cell types that are targeted by ENMs through common routes of exposure: lung epithelium, skin fibroblasts and adipocytes, gastrointestinal tract epithelia, and cells belonging to the reticuloendothelial system such as macrophages [4, 6]. Since all of these cells have unique functions in tissues and organs, there is no universal strategy for ENMs hazard assessment. As such, we should strive to develop appropriate assays for each cell type based on their specific functions. Another important aspect of ENMs safety evaluation lies in the understanding of how to distinguish between hazardous and safe concentrations and how to ensure that the proposed assays reveal a complete view of cellular changes induced by ENMs.

The current paradigm of ENMs hazard assessment is based on analysis of big data collected using high throughput screening (HTS) methods. However, the set of standard assays used for HTS assessment, only targets the symptoms of the cellular impairment such as decreased proliferation, changes in mitochondrial activity, or excessive reactive oxygen species (ROS) and lactate dehydrogenase (LDH). This approach allows for collection of secondary cellular responses to ENMs and limits our ability to detect earlier cell damage. The novelty of the proposed hazard assessment method is the detection of fundamental changes in early cell function. This approach is extremely sensitive and allows for detection of changes at earlier time prior to any damage detectable by the aforementioned standard assays. This approach also addresses proliferation and spreading of cells with impaired functions that might potentially cause the cascade of long term health problems. We believe that incorporating cell function based assays in the hazard assessment protocols will significantly reduce the number of long-term studies making the assessment process both, cost and time effective.

In this paper we use human ADSCs as a model to demonstrate that at low concentrations of ENMs, assays such as ROS, LDH, and cell proliferation, typically used for ENMs hazard assessment, reveal no damage. In contrast, other assays detect impairment of important cell functions. For this study we used two concentrations of TiO_2_ NPs, one that does not show an effect and another that significantly increases both ROS and LDH.

We chose TiO_2_ NPs for this study because of their abundance and availability. Previously, it was estimated that by 2015 more than 200,000 metric tons of TiO_2_ NPs will be manufactured annually [7]. We investigated two most abundant forms of TiO_2_, rutile and anatase, that have a tetragonal crystal structure but different atomic arrangement [8]. Considering, that these NPs are currently being used in products such as pharmaceuticals, personal care, cosmetics, toothpaste, sunscreens and food additives [9–11] it makes the possibility of human exposure guaranteed. Recently, the International Agency for Research on Cancer has classified the TiO_2_ particles as ‘‘*possibly carcinogenic to humans*’’ [12]. Hence, there is an urgent need for a proper environmental and health hazard assessment of these NPs with respect to concentration and route of exposure.

Human skin is in constant contact with the external environment and is one of the most important routes of exposure to TiO_2_. It is also the predominant organ exposed to high concentrations of TiO_2_ NPs used in sunscreens and personal care products [9, 11]. Even though, the latest FDA regulations of over-the-counter sunscreen products for human use allow up to 25% of TiO_2_ as an active ingredient, there are no regulations with regard to the size of the TiO_2_ or labeling of sunscreens for the inclusion of NPs [13]. Not surprising, recent tests of different sunscreens revealed TiO_2_ nanoparticles in each of them [14]. Similarly, tests performed by the Australian government showed that 70% of the sunscreens formulated with TiO_2_ in nanoparticulate form [15]. Studies from other countries also indicated that NPs are being used in sunscreen regardless of labeling [14, 16].

Adipose tissue is an abundant, accessible and rich source of adult stem cells suitable for tissue engineering and regenerative medicine. The ADSCs are adult mesenchymal stem cells that can be isolated from subcutaneous adipose tissue (bottom layer of skin) and have a preadipocyte characteristics [17]. They can also be induced to differentiate into adipocytes, bone marrow, neurons and other cell types [18–22]. The main function of these cells in their non-differentiated state is healing of cutaneous injuries while in their differentiated state, to contribute to fat depot maintenance [23].

Even though several research groups showed that nanosized TiO_2_ can penetrate through the skin in vivo [24, 25], the majority of reports showed that after application on skin, TiO_2_ NPs mostly reside in the stratum corneum and do not reach living skin [26, 27]. The 2013 Scientific Committee on Consumer Safety stated that the use of TiO_2_ NPs as a UV-filter in sunscreens, pose no adverse effects in humans when applied on healthy, intact or sunburnt skin [28]. Therefore, this manuscript aims to begin filling the knowledge gap that exists regarding the effects of TiO_2_ NPs on compromised skin (due to various diseases or trauma) which enables NPs to penetrate down to the subcutaneous adipose tissue layer. This presents a realistic scenario since exposure to sunlight is known to aggravate skin diseases such as psoriasis, dermatitis, eczema, and acne with daily application of sunscreen recommended [29, 30]. Moreover, TiO_2_ is a common ingredient in topical medications (ex. Sorion and Novasone cream) for the aforementioned conditions [31, 32] with a recommended daily application. Here, in addition to the standard ROS, LDH, and proliferation assays, we focused on the effects of TiO_2_ exposure on distinct functions of ADSCs: wound healing ability and intracellular lipid accumulation. Altogether, the current study indicates that specialized assays exhibit greater sensitivity in detecting damage induced by TiO_2_ NPs in ADSCs as compared to the current set of standard assays. Hence, we propose that for proper ENMs assessment, the standard set of assays needs to be expanded and include tests examining the impairment of characteristic cell functions. Moreover, such approach may establish appropriate guidelines and identify safe concentrations of ENMs.

## Methods

Anatase and Rutile TiO_2_ NPs were purchased from US Cosmetics. Trypsin–EDTA (0.05%) (Cat#: 25300-054) and Dulbecco’s Phosphate-Buffered Saline (Cat#: 14190-250) were purchased from Life Technologies. Alexa Fluor 488-Phalloidin (Cat#: A12379), LipidTOX™ red (Cat#: H34476) were purchased from ThermoFisher Scientific.

### Cell culture

Primary human ADSCs were obtained from Living Skin Bank (Stony Brook University) and were cultured in basal medium comprising Dulbecco’s Modified Eagle’s Medium (DMEM) supplemented with 10% fetal bovine serum (FBS; HyClone, Logan, UT) and 1% of penicillin–streptomycin (PS; Sigma, St. Louis, MO). For differentiation, ADSCs were cultured in basal medium supplemented with 250 μM 3-isobutyl-1-methylxanthine (IBMX), 1 μM insulin, 200 μM indomethacin, 33 μM biotin, 17 μM pantothenic acid and 1 μM dexamethasone (adipose induction medium). Medium containing TiO_2_ NPs (with concentrations of 0.1 and 0.4 mg/mL) was added to each culture plate 24 h after initial cell seeding. The samples were incubated with NPs up to 6 days and then counted or fixed, stained and imaged. Culture medium was replaced every 2–3 days and grown at 37 °C with 5% CO_2_. NP-free cultures served as controls.

### Cell proliferation

To determine cell proliferation, cells were plated at an initial density of 7.5 × 10^3^ cells per well in DMEM supplemented with 10% FBS and 1% of PS in a 12-well tissue culture plate and counted using a hemocytometer at days 1, 2, 3, and 6. Each condition was completed in triplicates (n = 3) and all experiments were conducted three times (n = 3). Medium was changed every 2 days.

### Zeta potential and dynamic light scattering

To prepare the samples, 2 µg of TiO_2_ NPs were placed in 10 mL of deionized water or culture medium and sonicated for 5 min to separate agglomerates. The samples were then diluted ten times in deionized water, briefly sonicated and analyzed. Zeta potentials were measured using Brookhaven Instruments Zeta Plus Zeta Potential Analyzer and particle size measurements were performed using BIC 90Plus dynamic light scattering (DLS) instrument (Brookhaven Instruments, Zeta Plus Zeta Potential Analyzer). The average of 3 measurements of 50 cycles was used as a numerical value of zeta potential.

### Transmission electron microscopy (TEM)

TEM analysis was used to assess the size and distribution, as well as intracellular localization of TiO_2_ NPs. For the particle analyses, one drop of TiO_2_ NPs suspension was placed on a 300 mesh Formvar coated copper grid and air dried at room temperature. A histogram of the size distribution from approximately 170–200 particles was plotted and fit to a Gaussian distribution from which the mean diameters were obtained.

For intracellular particles distribution, 1 × 10^5^ cells per well were plated in six-well plate, exposed to TiO_2_ NPs for 3 days and then fixed in a solution of 2.5% paraformaldehyde and 2.5% glutaraldehyde in 0.1 M Phosphate Buffered Saline (PBS). The samples were then dehydrated with ethanol and embedded in propylene oxide. The specimens were then cut into ultrathin (90 nm) sections using a Reichart Ultracut Ultramicrotome, lifted onto uncoated TEM grids and stained with uranyl acetate and lead citrate. The samples were imaged using a FEI Tecnai12 BioTwinG2 transmission electron microscope. Digital images were acquired with an AMT XR-60 CCD Digital Camera System.

### Delivered and cellular TiO_2_ NPs doses

The theoretical estimation of delivered doses (mass of TiO_2_ NPs deposited per area) was performed using in vitro sedimentation, diffusion and dosimetry (ISDD) model generously provided by Dr. Teeguarden [33]. This computational model of particokinetics (sedimentation, diffusion) estimates the amount of particles reaching cells residing at the bottom of a cell culture dish during a defined exposure period. The model also calculates fraction of particles, surface area, mass and number of particles reaching cells and allows comparison of particle doses among particle types within a system, and among systems with different characteristics (media height, viscosity, orientation).

### Effective density by volumetric centrifugation method (VCM)

Effective density of the TiO_2_ NPs agglomerates was estimated using VCM adopted from Deloid et al. [34]. Briefly, a sample of TiO_2_ NPs suspension in basal medium was centrifuged in a packed cell volume (PCV) tube (Sigma Aldrich, Cat#: Z760986) at 3000 rpm for 1 h to produce a pellet consisting of packed NPs agglomerates and the media trapped between them. All VCM experiments were performed in triplicates (n = 3) and the average was used to calculate the effective agglomerate density.

The effective density of the TiO_2_ NPs agglomerates, *ρ*_EV_, was calculated using following equation [34]:

1 $$ \rho_{EV} = \rho_{media} + \left[ {\left( {\frac{{M_{{TiO_{2} }} }}{{V_{pellet} \cdot SF}}} \right) \cdot \left( {1 - \frac{{\rho_{media} }}{{\rho_{{TiO_{2} }} }}} \right)} \right] $$

Where *ρ*_TiO2_ is TiO_2_ NPs density, *M*_TiO2_ is mass of TiO_2_ NPs, *V*_pellet_ is the volume of the pellet collected by centrifugation, *ρ*_media_ is the media density, and SF is stacking factor that depends on the efficiency of agglomerate stacking. In this paper, we used theoretical *SF* values of 0.634 for anatase and 0.7 for rutile as it was previously recommended as a reasonable approximation by DeLoid et al. [34].

### Cell staining for confocal microscopy

Cell area and overall morphology as a function of NP uptake was monitored using a Leica confocal microscope. For these experiments, cells were exposed to TiO_2_ for 3 weeks of differentiation and then fixed with 3.7% formaldehyde for 15 min. Alexa Fluor 488-Phalloidin was used for actin staining and lipid droplets were visualized using LipidTOX™ red according to the manufacturer’s instructions.

### Lactate dehydrogenase activity (LDH) measurements

Pierce LDH Cytotoxicity Assay Kit (Cat#: 88953, Life Technology) was used for LDH measurements. Cells were plated with starting density of 8 × 10^4^ per well in six-well plate. After 3 days of incubation with nanoparticles, 50 μL supernatant from each sample were transferred to a 96-well plate in triplicate wells and 50 μL of reaction mixture (lyophilizate mixture) were added. After incubation at room temperature for 30 min, the reaction was stopped by adding 50 μL Stop Solution. Released LDH activity absorbance was measured at 490 and 630 nm respectively.

### Reactive oxygen species (ROS) measurement

ROS Detection Reagents (Cat#: C6827, Invitrogen) was used to detect ROS level of ADSCs cells. For this experiment a working solution of 5 µg/mL of 5-(and-6)-chloromethyl-2′,7′-dichlorodihydrofluorescein diacetate, acetyl ester (CM-H_2_DCFDA) was prepared. Cultures were seeded with starting density of 8 × 10^4^ per well in six-well plate and exposed to TiO_2_ for 3 days. Cells were then harvested and washed three times with PBS to remove TiO_2_ NPs from pellets, counted and 5 × 10^4^ cells per well were placed to 96-well dish (each condition had triplicates). Then 100 µL of working solution was added to each well and incubated for 20 min. 100 µL of 20 mM NaN_3_ were then added to each well and incubated for 2 h. Fluorescence was read at 490 nm excitation and 520 nm emission.

### Migration

Cell migration of cultures seeded at 8 × 10^4^ cells per well in six-well plate and treated with TiO_2_ NPs for 3 days was evaluated using the agarose droplet assay. The agarose gel was prepared by melting a 2% (w/v) agarose stock solution, and diluting it with DMEM to 0.2% (w/v). The 0.2% (w/v) agarose was then used to re-suspend cells to a concentration of 1.5 × 10^7^ cells/mL. After that 1.25 µL drops were placed into each well of a 24-well dish, and allowed to gel at 4 °C for 20 min prior to the addition of 400 μL of DMEM into each well. Following a 24 h incubation at 37 °C, the cells were visualized under phase contrast microscopy. Cell migration from the outer edge of the agarose was quantified using imageJ software.

### Collagen gel contraction

Cells seeded at initial density of 8 × 10^4^ per well in six-well plate were exposed to 0.1 and 0.4 mg/mL TiO_2_ NPs for 3 days. After that cultures were harvested and resuspended in DMEM containing 1.8 mg/mL collagen and 2% BSA at 3.5 × 10^5^ cells/mL. Cell/collagen gel suspensions (0.7 mL) were loaded into each well of 24-well dish pre-coated with 2% BSA in PBS coated (overnight) and incubated at 37 °C to induce gelation. After 2 h the gel was detached by tapping lightly on the wall of the wells and 500 μL DMEM with 2% BSA was added. Detachment was done in order to begin the contraction process. The gels were then incubated for 5 h and imaged by scanning the 24 well plate.

### Lipid quantification and visualization

To determine differences in lipid accumulation, cells were differentiated for 1, 2, and 3 weeks in adipose induction media were fixed with 3.7% formaldehyde for 15 min at room temperature and incubated with Oil red O for 2 h. Oil red O was then extracted using isopropanol and the amount of lipids was measured as a function of Oil red O absorbance (510 nm). Lipid amounts were calculated on a per cell basis, where a typical sample contained 1.5–2.0 × 10^5^ million cells per well. The cellular distribution of lipid droplets was visualized using confocal microscopy as described below.

### Adiponectin expression

Cell culture media was collected on days 7, 14 and 21 after switching to adipose induction media. The cells were stored at −20 °C in the presence of a protease inhibitor cocktail (Cat#: P8340, Sigma-Aldrich) till assay time. Adiponectin was directly measured using the human adiponectin ELISA kit (Novex^®^, Cat#: KHP0041). Samples were prepared according to manufacturer’s instructions and the absorbance was read at 450 nm using a microplate reader (BioTek EL800).

### Collagen and fibronectin expression

Collagen and fibronectin in the cell culture media of ADSCs cultured for 3 days was measured using the Procollagen Type I C-Peptide EIA Kit (Takara) and human Fibronectin EIA Kit (Takara) as described in instructions provided by the manufacturer. Samples with high concentrations of collagen and fibronectin were diluted with Sample Diluent (Takara) prior to assay. Absorbance was read at 450 nm using a microplate reader (BioTek EL800).

### Flow cytometry

Cell were plated, allowed to adhere for 24 h, and exposed to 0.1 and 0.4 mg/mL TiO_2_ nanoparticles for another 3 days. The cells were carefully rinsed with PBS three times to remove all the floating particles and were detached by gentle scrapping. The cells were then rinsed twice with BSA (0.2%) in PBS and re-suspended in PBS at a concentration of 10^6^ cells/mL. All samples were then analyzed with a Becton–Dickinson FACSCAN analyzers flow cytometer.

### Statistical analysis

All experiments were performed in triplicates and repeated at least three times. The results were represented as mean ± SD. A p value <0.05 was considered statistically significant (t test).

## Results

### Characterization of TiO_2_ NPs

**Fig. 1:**
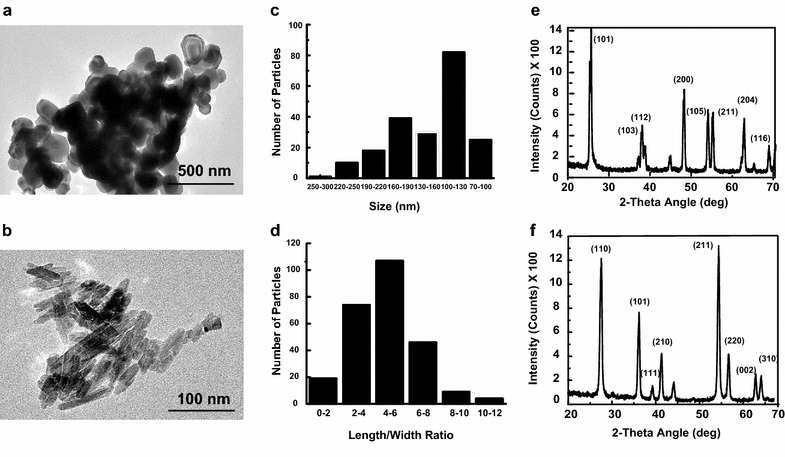
TiO_2_ nanoparticles imaged by TEM, its size distribution histograms and X-ray diffraction spectra. TEM picture of anatase **a** nanoparticles and rutile, **b** TiO_2_ nanorods; size distribution histograms of anatase (**c**) and rutile (**d**); X-ray diffraction spectra of anatase (**e**) and rutile (**f**)

Anatase particles have a spherical shape, while rutile particles are rod shape with the aspect ratio of 4 (Fig. 1a, b). From the TEM images, the calculated average diameter of anatase is 136 ± 47 nm and the average length of rutile is 46 ± 28 nm (Fig. 1c, d). X-ray diffraction spectra of both particles are shown on Fig. 1e, f confirming anatase and rutile crystal structures. The surface charges of the particles were measured using zeta potentiometry (Table 1), and were found for particles suspended in deionized water to be −34.75 ± 1.63 and −30.29 ± 0.6 mV for anatase and rutile respectively. After NPs incubation in DMEM for at least 24 h their zeta potential increased to −7.39 ± 0.90 and −14.29 ± 1.73 mV for anatase and rutile respectively. Surface charge of TiO_2_ NPs suspended in adipose induction media was −13.85 ± 0.9 and −12.45 ± 2.3 mV for rutile and anatase, respectively.

**Table 1:** Properties of TiO_2_ NPs

	Anatase	Rutile
Zeta potential (mV)		
In DI water	−34.75 ± 1.63	−30.29 ± 0.6
In DMEM	−7.39 ± 0.90	−14.29 ± 1.73
In induction media	−13.85 ± 0.9	−12.45 ± 2.3
DLS (nm)		
In DI water	383 ± 19	640 ± 44
In DMEM	355 ± 37	291 ± 37
In induction media	368 ± 37	408 ± 38
Density of agglomerates (g/mL)		
In DMEM	2.428	3.383
Volume of pellets (µL)		
In DMEM	0.486 ± 0.076	0.766 ± 0.052

DLS was performed to determine the hydrodynamic sizes of the TiO_2_-NPs in suspension assuming that particles are spherical or can be represented as spheres (average number distributions are reported, Table 1). The dispersions of rutile and anatase TiO_2_-NPs were homogeneous and only revealed the presence of secondary aggregates. Anatase TiO_2_ NPs have aggregates of 383 ± 19 nm in water, 355 ± 37 nm in basal medium and 368 ± 37 nm in adipose induction media. Similarly, aggregates of rutile in water, basal and induction media were 640 ± 44, 291 ± 37, and 408 ± 38 nm, respectively.

### Delivered doses of TiO_2_ NPs

For proper assessment of TiO_2_ NPs the actual delivered doses that come in contact with cells were estimated using the ISDD model (Table 1). The densities of TiO_2_ agglomerates were calculated using Eq. 1 (see “Methods”) and were 2.428 and 3.383 g/mL for rutile and anatase, respectively. Density of basal media was 1.007 g/mL [33] and volumes of the anatase and rutile pellets, measured by volumetric centrifugation, were 0.486 ± 0.076 and 0.766 ± 0.052 µL, respectively. Finally, delivered fractions of TiO_2_ NPs after 72 h of incubation were 1 for both anatase and rutile, making the corresponding delivered doses equal to 10 and 40 µg/cm^2^ for initial 0.1 and 0.4 mg/mL treatments, respectively. In fact, according to these calculations, 100% of particles were deposited on cells after the first 16 h of incubation.

### Proliferation of ADSCs

**Fig. 2:**
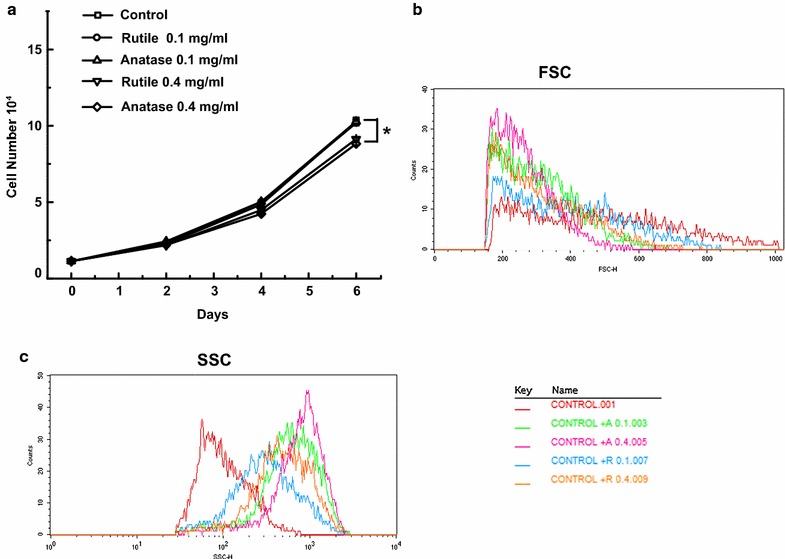
**a** Proliferation of ADSCs exposed to 0.1 and 0.4 mg/mL anatase and rutile TiO_2_ for 6 days and control unexposed cells. **b** Forward-scattered light (FSC) data of ADSCs after exposure for 3 days to 0.1 and 0.4 mg/mL anatase and rutile TiO_2_ and unexposed control. **c** Side-scattered light (SCC) data of ADS cells after exposure for 3 days to 0.1 and 0.4 mg/mL anatase and rutile TiO_2_ and unexposed control

In order to directly observe the impact of TiO_2_ NPs exposure on the ADSCs, we measured cell proliferation after incubation with TiO_2_ NPs for up to 6 days. The data obtained from counting the cells is shown in Fig. 2. It is apparent that exposure to low concentration (0.1 mg/mL) of both rutile and anatase NPs had no effect on cell proliferation (Fig. 2a). In contrast, exposure to 0.4 mg/mL resulted in a moderate decrease in cell number after prolonged exposure. Specifically, on day 4 we observed approximately 11 ± 1% decrease in after exposure to anatase that extended to 16 ± 2% decrease on day 6. In case of ADSCs exposed to rutile, the decrease was approximately 9 ± 1 and 14 ± 1% on days 4 and 6, respectively. The decrease in cell numbers observed after 6 days of treatment with 0.4 mg/mL TiO_2_ was statistically significant.

### Internalization of TiO_2_ NPs

Flow cytometry was used to measure cell granularity and confirm internalization of TiO_2_ NPs by ADSCs. From Fig. 2b, c we can see an increase in side scatter (SSC) and the slight decrease in forward scatter (FSC) in all cultures treated with TiO_2_ NPs. Also, SSC increased in a NPs dose dependent manner, the overall observed increase was higher for anatase than rutile using identical concentrations.

**Fig. 3:**
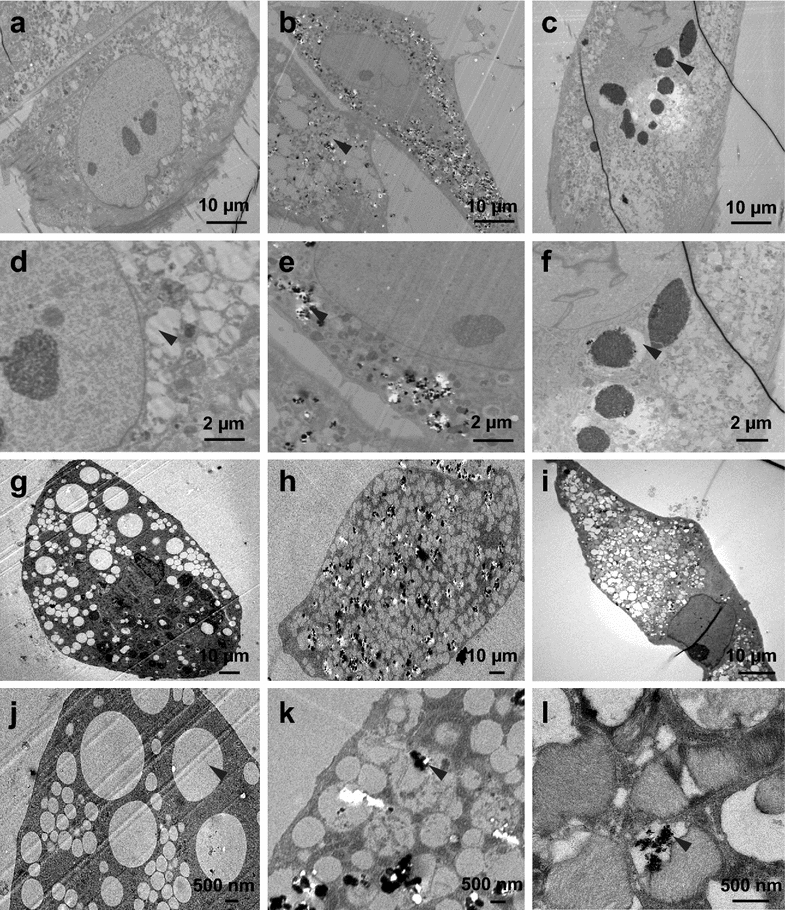
TEM cross section of ADSCs exposed to TiO_2_ NPs in different conditions: in basal media (**a**–**f**), after 3 weeks in adipose induction media (**g**–**l**). ADSC control cells in basal media (**a**), ADSCs exposed to 0.4 mg/mL anatase (**b**) and 0.4 mg/mL rutile (**c**) for 3 days in basal media. ADSC control in basal media high magnification (**d**), ADSCs exposed to 0.4 mg/mL anatase (**e**) and 0.4 mg/mL rutile (**f**) for 3 days in basal media high magnification. ADSC control cells in induction media (**g**) for 3 weeks, ADSCs exposed to 0.4 mg/mL anatase (**h**) and 0.4 mg/mL rutile (**i**) for 3 weeks. ADSC control cells in induction media high magnification (**j**), ADSCs exposed to 0.4 mg/mL anatase (**k**) and 0.4 mg/mL rutile (**l**) in induction medium for 3 weeks high magnification. *Black arrows* indicate the vacuoles (**a**–**f**) and lipid droplets (**g**–**l**)

For accurate characterization of NP-cell interaction it is important to know the localization of NPs within cells. Figure 3 shows TEM images of ADSCs that had been cultured with rutile or anatase NPs for 3 days in basal media or for 3 weeks in adipose induction media. Figure 3a–f shows that after incubation in basal media, both types of TiO_2_ NPs accumulated in vacuoles. However, the size of the vacuoles in which rutile and anatase TiO_2_ NPs are stored is significantly different. Rutile-containing vacuoles were 3.2 ± 1.4 µm which is approximately ninefold larger that those filled with anatase (0.29 ± 0.2 µm) (Fig. 3a–c). In addition, the shape of the rutile containing vacuoles is mostly spherical with well-defined edges as oppose to the anatase containing vacuoles that display irregular shape without distinct edges (Fig. 3d–f). Also, inside the vacuole, rutile particles seem to be tightly packed whereas with anatase they are more loose. Cellular compartments such as nuclei, mitochondria, rough endoplasmic reticulum (ER), and Golgi apparatus did not contain TiO_2_ NPs.

Images of ADSCs after differentiation for 3 weeks in adipose induction media containing TiO_2_ NPs are shown on Fig. 3g–l. All the TEM images reveal adipose conversion (lipid droplet formation) in cultures treated with TiO_2_ NPs as well as control. However, significantly smaller amounts of TiO_2_ NPs can be found inside the cells (Fig. 3j–l). Once again, nuclei, mitochondria, rough ER, and Golgi apparatus were devoid of TiO_2_ NPs. However, we did observe that both rutile and anatase NPs penetrate lipid droplets as can be seen on Fig. 3k, l as indicated by the arrowheads. It is interesting to note, that TiO_2_ NPs can percolate the phospholipid monolayer that surrounds lipid droplets, however they are unable to penetrate through the phospholipid bilayer of mitochondria, nuclei, endoplasmic reticulum, and golgi apparatus. Additionally, such structural disturbance of lipid droplets may potentially cause leakage of lipids interfering with fat storage and metabolism.

### TiO_2_ NPs cytotoxicity

**Fig. 4:**
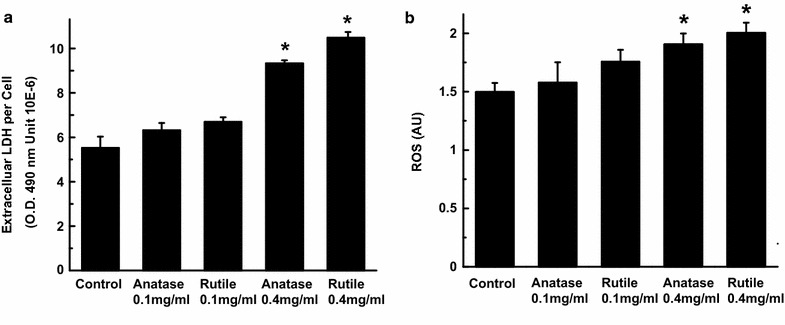
**a** Extracellular lactate dehydrogenase amount in ADSC control and culture exposed to 0.1 and 0.4 mg/mL anatase and rutile TiO_2_ for 3 days. **b** ROS generated by ADSCs after 3 days exposure to 0.1 and 0.4 mg/mL anatase and rutile TiO_2_ and unexposed control

Even though no major changes in cell proliferation were observed with both types and concentrations of TiO_2_ NPs for short (3 days) exposure, secondary processes triggered by the exposure such as secretory or morphological changes may induce long-term toxicity. To test this, we investigated two of the most common indicators of ENMs cytotoxicity: cellular ROS generation and LDH release. In Fig. 4a we show that no increase in ROS was observed in the cultures treated with 0.1 mg/mL rutile and anatase NPs. On the other hand, cells exposed to 0.4 mg/mL TiO_2_ NPs for 3 days exhibited 27 ± 5 and 33 ± 4% increase in ROS levels after treatment with rutile and anatase, respectively.

Release of LDH is associated with the loss of cell-membrane integrity and is another important indicator of cellular toxicity induced by ENMs. A recent study reported that LDH binds to TiO_2_ NPs decreasing the assay readout [35], however concentrations of rutile and anatase used in our experiments were below the minimum concentration at which differences in LDH readout could be detected. From the Fig. 4b we can see that there are no changes in the extracellular levels of LDH in case of exposure to 0.1 mg/mL rutile and anatase as compared to control culture. In contrast, cultures exposed to 0.4 mg/mL dose of rutile TiO_2_ NPs exhibit a ~33% increase in extracellular LDH while cultures treated with anatase show an LDH increase of ~43%.

### Cell migration and collagen gel contraction

**Fig. 5:**
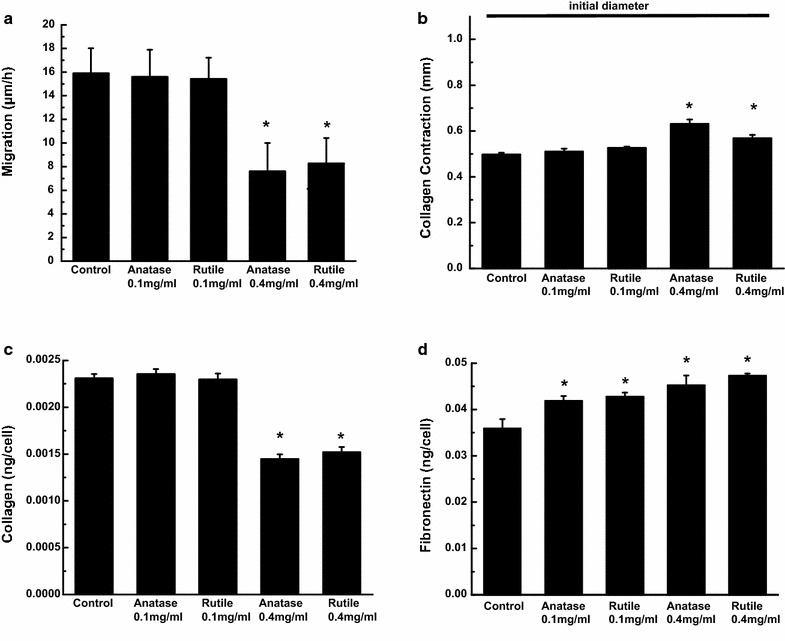
ADSCs migration (**a**), collagen contraction (**b**), ECM collagen secretion (**c**) and ECM fibronectin secretion (**d**) after the exposure to 0.1 and 0.4 mg/mL anatase and rutile TiO_2_ NPs for 3 days

One of the crucial functions of ADSCs is their ability to migrate into a wound and contract newly deposited collagen fibers to close the wound. Hence, we examined this function after ADSCs exposure to TiO_2_ NPs for 3 days. As shown in Fig. 5a, b, exposure to 0.1 mg/mL TiO_2_ NPs had no effect on the cell migration speed as well as their ability to contract collagen. However, exposure to 0.4 mg/mL NPs significantly altered both functions. Specifically, it yielded a 15 ± 2 and 27 ± 3% decrease in collagen contraction in ADSCs treated with rutile and anatase, respectively (Fig. 5a, b). A similar trend was observed in cell migration speed; decrease in speed by 48 ± 13 and 52 ± 15% in cultures exposed to 0.4 mg/mL rutile and anatase, respectively.

### Changes in ECM

It is known that extracellular matrix (ECM) proteins play a crucial role in cell behavior and regulate important cellular functions such as proliferation, apoptosis, and migration [36]. Thus, we decided to examine whether exposure of ADSCs to TiO_2_ NPs affects two major ECM components, collagen type I and fibronectin. Figure 5c shows that there is no change in collagen expression in cultures exposed to low concentrations of rutile and anatase as compared to control. On the other hand, exposure to 0.4 mg/mL of rutile and anatase TiO_2_ leads to a reduction of collagen by 35 ± 5 and 37 ± 4%, respectively. Interestingly, we did not observe a similar pattern in fibronectin production. Figure 5d indicates that exposure of ADSCs to increasing doses of TiO_2_ results in small, but steady increase in fibronectin as compared to control. Specifically, our data indicates that the fibronectin concentration increased by 17 ± 2 and 19 ± 2% in cultures treated with 0.1 mg/mL TiO_2_ and by 26 ± 5 and 32 ± 1% in cultures treated with 0.4 mg/mL for anatase and rutile, respectively.

### Adipocyte-specific differentiation

Another important function of ADSCs is their ability to differentiate into adipocytes. Given the appropriate signals ADSCs will differentiate into adipocytes and accumulate lipid droplets as well as express adipocyte specific proteins. Therefore, we investigated the effect of TiO_2_ NPs on lipid accumulation and the expression of an adipocyte differentiation marker adiponectin that is involved in several metabolic pathways including glucose and fatty acid catabolism [37]. Experiments were conducted using cultures exposed to rutile and anatase TiO_2_ NPs for 3 weeks during adipocyte induction.

#### Lipid accumulation

**Fig. 6:**
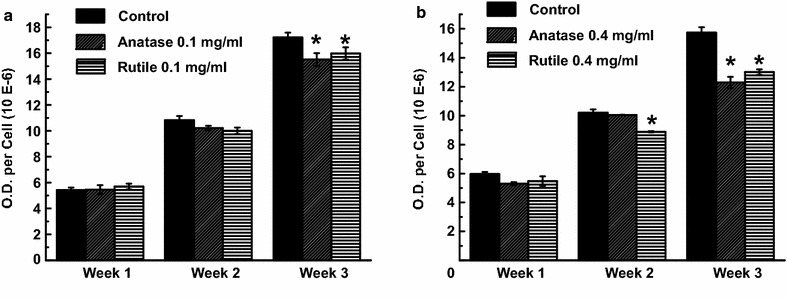
Lipid accumulation in ADSCs differentiated for 3 weeks in the presence of 0.1 mg/mL (**a**) and 0.4 mg/mL (**b**) anatase and rutile TiO_2_ NPs

**Fig. 7:**
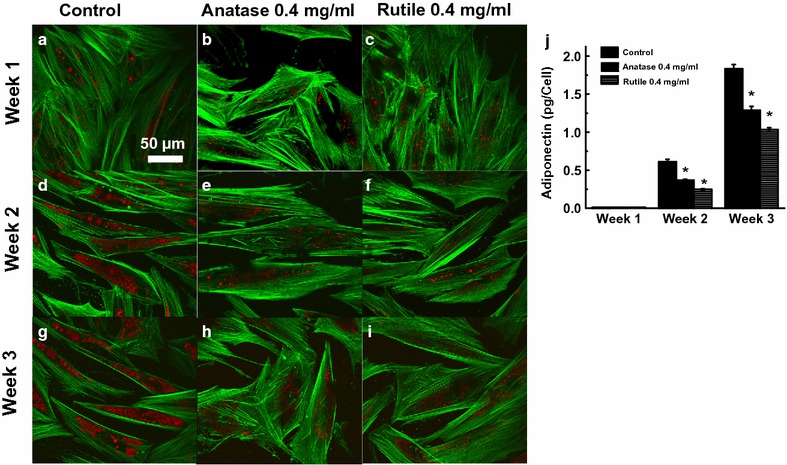
Confocal microscopy images of ADSCs differentiated for 3 weeks in the presence of 0.4 mg/mL anatase and rutile TiO_2_ NPs (**a**–**i**). ADSCs were stained for actin filaments (*green*) and lipid droplets (*red*). ADSCs control differentiated for 1 weeks (**a**), ADSCs differentiated for 1 weeks with 0.4 mg/mL anatase (**b**) and rutile (**c**) TiO_2_ NPs. ADSCs control differentiated for 2 weeks (**d**), ADSCs differentiated for 2 weeks with 0.4 mg/mL anatase (**e**) and rutile (**f**) TiO_2_ NPs. ADSCs control differentiated for 3 weeks (**g**), ADSCs differentiated for 1 weeks with 0.4 mg/mL anatase (**h**) and rutile (**i**) TiO_2_ NPs. **j** Adiponectin secretion after 1, 2 and 3 weeks of differentiation with 0.4 mg/mL anatase and rutile TiO_2_ NPs and control

Lipid accumulation was examined in ADSCs grown in NP-containing induction medium where the NP-free cultures served as control. From Fig. 6a we can see that cultures exposed to 0.1 mg/mL NPs had the same quantity of lipids as control after 1 and 2 weeks of exposure. A small decrease in lipids accumulation of 10 ± 3 and 12 ± 3% was detected after 3 weeks in cultures treated with rutile and anatase (0.1 mg/mL), respectively. Higher reduction in lipids was observed in ADSCs exposed to 0.4 mg/mL TiO_2_ NPs; exposure to 0.4 mg/mL rutile resulted in 11 ± 1% decrease after 2 weeks of exposure and extended to 15 ± 1% by the end of week 3 (Fig. 6b). Reduction in lipids in cultures treated with 0.4 mg/mL anatase was 22 ± 3% after 3 weeks (Fig. 6b). These data correlate with our qualitative observation via confocal microscopy, where reduction in lipid droplet size was seen in both cultures grown with TiO_2_ NPs for 3 weeks (Fig. 7g–i); and only cultures exposed to rutile showed decrease in lipids after 2 weeks. No changes were seen at an earlier time point (Fig. 7a–f).

#### Adipocyte-specific adiponectin secretion

To determine whether TiO_2_ NPs affect the expression of adipocyte specific cytokines, we measured adiponectin concentration in cultures grown in induction medium containing 0.4 mg/mL TiO_2_. As expected, after 1 week in induction medium no adiponectin was detected in either control or treated cultures (Fig. 7j). By week two of ADSC differentiation, control cultures showed an increase in adiponectin secretion to 0.61 pg/cell and by week three, secretion reached 1.83 pg/cell. Smaller increases at week two were seen in the cultures incubated with TiO_2_ NPs, where adiponectin levels were 40 ± 3 and 59 ± 4% less than control in the cultures exposed to anatase and rutile, respectively. At week three, adiponectin concentration further increased in all cultures but resulted in levels that were 30 ± 4 and 42 ± 3% less than control, for those treated with anatase and rutile TiO_2_ NPs, respectively.

### Changes in ECM during differentiation

**Fig. 8:**
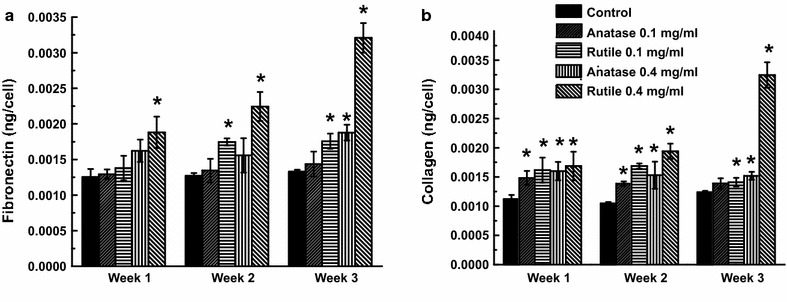
ECM fibronectin (**a**) and collagen (**b**) after the exposure to the 0.1, 0.4 mg/mL anatase and rutile nanoparticles for 3 days in basal media and then switched for differentiation in induced media for 3 weeks

In addition to proliferation and apoptosis regulation, ECM proteins are also involved in cell differentiation and lipid formation [38, 39]. Data in Fig. 8a reveals that cultures exposed to 0.1 mg/mL TiO_2_ NPs for 1 week while differentiating show no changes in fibronectin production as compared to control. Exposure of ADSCs to 0.4 mg/mL TiO_2_ increased fibronectin by 30 ± 10 and 50 ± 12% for anatase and rutile, respectively. Similarly, after 2 and 3 weeks of differentiation, no changes in fibronectin expression were observed for cultures treated with 0.1 mg/mL anatase. In contrast, exposure to 0.1 mg/mL rutile resulted in 40 ± 3% increase in fibronectin after 2 and 3 weeks of differentiation. In all cases, cultures exposed to higher doses of TiO_2_ exhibited substantial increase in fibronectin expression as compared to control cultures. Specifically, treatment with 0.4 mg/mL TiO_2_ NPs increased fibronectin production by 30 ± 10 and 50 ± 12% after 1 week, by 25 ± 14 and 79 ± 9% after 2 weeks, and by 50 ± 6 and 150 ± 7% after 3 weeks for anatase and rutile, respectively.

The effect of TiO_2_ NPs on the expression of collagen is shown on Fig. 8b. Data shows that after 2 week treatment, all cultures exposed to rutile and anatase, at both concentrations, have elevated collagen production. Cultures exposed to 0.1 mg/mL anatase and rutile exhibited an increase in collagen by 32 ± 8 and 44 ± 13%, respectively, while exposure to 0.4 mg/mL led to increases by 42 ± 10 and 50 ± 15%, respectively. After 2 weeks of adipose induction cultures exposed to TiO_2_ showed a further increase in collagen, ranging from 34 ± 3 to 61 ± 3% in cultures treated with low concentrations and by 48 and 87% in cultures treated with high concentrations of anatase and rutile, respectively. After 3 weeks of adipogenic induction the increase in collagen in cultures treated with 0.1 mg/mL rutile and anatase was approximately 12%. Cultures treated with 0.4 mg/mL anatase had an increase in collagen by 23 ± 5% as compared to control TiO_2_ untreated cultures. Interestingly, the increase in collagen in culture treated with 0.4 mg/mL rutile TiO_2_ after 3 weeks of differentiation was 162 ± 7% relative to control untreated cultures (Fig. 8b).

## Discussion

Increased production of TiO_2_ NPs and their application in a variety of consumer products require careful dose and hazard assessment if we are to establish a concentration range that ensures safety for human health and the environment. The first step in hazard assessment should be proper characterization of ENMs in “as synthesized” state (e.g. in water) and, more importantly, in relevant environment or “as administrated” (e.g. in cell media for in vitro experiments). This will help to obtain meaningful and reproducible results and correlate ENMs properties with their potential toxicity in vitro [2, 5].

In this paper, we explored the range of TiO_2_ NPs concentrations by testing one concentration that has no effect on cellular behavior based on a standard set of toxicity assays (ROS, LDH, and proliferation) and with another concentration that demonstrates their hazardous potential. In addition to standard assays, we performed tests addressing the TiO_2_ NPs effects on cell function that are characteristic for the chosen model cell line. We successfully demonstrated that for sufficient safety assessment of NPs in ADSCs cultures these assays are necessary as they exhibit greater sensitivity and may be used for identifying the guideline for safe concentrations.

The TiO_2_ NPs chosen for this study have chemically unmodified negatively charged surface. It is known that the adsorption of proteins on the surface of the NPs changes the overall particle charge. Both basal and adipose induction medium used in this study were supplemented with 10% FBS that contains significant amount of albumin protein which has high affinity toward TiO_2_ [40, 41]. As a result, we observed that protein corona adsorbed on the surface significantly increased the surface charge of both rutile and anatase. These findings are in agreement with previous reports by several research groups who showed that decrease in the surface charge of TiO_2_ NPs results from adsorption of albumin and apolipoproteins from media supplemented with FBS [40, 42, 43]. Such a protein corona plays a critical role in NP stability and in the absence of albumin TiO_2_ NPs sediment rapidly, as opposed to stable suspension that forms in the presence of albumin [43, 44]. Similarly, in our experiments all media-based TiO_2_ NPs suspensions exhibited prolonged stability as compared to water-based suspensions.

Since TiO_2_ NPs are suspended in basal or adipose induction medium prior to ADSCs treatment, it is important to determine the size of their aggregates. Therefore, to identify these parameters for in vitro cytotoxicity, we performed TiO_2_ NP aggregation assessment by DLS. This is a standard method used to measure the Brownian size of NPs in colloidal suspensions. The average hydrodynamic diameter (secondary TiO_2_ NPs size) of anatase aggregates was similar in all conditions revealing small aggregates of 2–3 particles each. On the other hand, aggregates of rutile in both, basal and induction media were determined to be smaller as compared to suspensions in Milli-Q water, indicating the stabilizing effect of the media due to the protein adsorption on the particle surface. This is in agreement with previously reported findings by multiple research groups [45–48].

Once suspended in liquid, TiO_2_ NPs form fractal agglomerates [48, 49] decreasing the total number of free particles, and as a result, the total surface area available for bio-interactions [34]. Our measurement of TiO_2_ NPs “effective density” confirmed that agglomerates are porous, and contain media trapped inside [33, 49] as their density was significantly smaller than the density of primary particles. Such findings are in alignment with previously published assessment of NPs [34] where authors demonstrated a similar trend; effective density of various NPs agglomerates in culture media is decreased as compared to the density of the material.

Since cells only respond to the NPs they come in contact with, we calculated the actual dose of NPs causing the effect—“delivered dose of NPs”. It is known that rates of NP sedimentation, diffusion and agglomeration depend not only on their size, density, but also on surface physicochemistry and media characteristics [50]. All these properties determine the transport of NPs and significantly affect the delivered dose. Recently, a computational model of particokinetics and dosimetry for non-interacting spherical particles has been developed [33]. This ISDD model accounts for Stokes sedimentation and Stokes–Einstein diffusion and was demonstrated to have accurate predictions for particles of different sizes and densities [33]. Our calculations indicated that after 19 h of exposure 100% of administrated TiO_2_ NPs mass comes in contact with cells. Therefore, we speculate that the observed toxicological response of the ADSCs treated with 0.1 and 0.4 mg/mL TiO_2_ is equivalent to the response to 10 and 40 µg/cm^2^ of TiO_2_ NPs applied for 56 h in an in vivo study. It is important to note that according to FDA recommendations the amount of sunscreen that needs to be applied on the skin to achieve labeled SPF rating is 2 mg/cm^2^ [51]. Since the FDA approves 25% by weight of TiO_2_ in sunscreens [13], the amount of TiO_2_ NPs in contact with skin can be as high as 0.5 mg/cm^2^. Hence, the TiO_2_ doses tested in this study are more than tenfold smaller than what is approved by FDA and thus reflects a real life scenario. Moreover, the FDA recommends re-application of the sunscreen every 2 h, in this case during 8 h of exposure (outdoor workers or sunbathers) the amount of TiO_2_ NPs in contact with skin can reaches 4 mg/cm^2^. In addition, since sun exposure as well as medical treatment of skin diseases is likely to be a continuous process requiring at least daily application of sunscreen or medicine for a prolonged time, the exposure time used in our study is certainly representative.

Various research groups reported that TiO_2_ NPs aggregates enter cells mainly via endocytosis and reside in the vacuoles and cell cytoplasm [52, 53] Interestingly, in our study, the mechanism of intracellular sequestration appears to be different for rutile and anatase TiO_2_ NPs. Close inspection of TEM images reveals that rutile NPs are stored in a few large vacuoles (~2.7 µm) within the cytoplasm, whereas anatase NPs are sequestered in great number of much smaller vacuoles (~290 nm) that distributed uniformly across the cytoplasm. Also, similar to previous findings in other cell types [53, 54], we found that TiO_2_ NPs did not enter the nucleus or any other organelles. However, TiO_2_ NPs penetrated lipid droplets in the differentiated ADSCs, raising the question whether such disruption can cause lipid leakage and contribute to the overall decrease in intracellular lipid accumulation. Similar case of TiO_2_ NPs penetrating lipids was reported in the in vivo study where authors observed that TiO_2_ NP penetrated oil storage droplets in water flea *Daphnia magna* [55]. Similar to our case, these storage cells mainly contain lipids such as triacylglycerol that are used by *D. Magna* in periods of low food resources. In another report analogous changes were observed in the terrestrial isopod *Porcellio scaber* exposed to TiO_2_ NPs [56]. Lastly, it was recently reported that TiO_2_ NPs have an affinity to triglycerides and easily absorb them on their surface [57].

The cellular uptake of TiO_2_ NPs was confirmed by cell granularity measurements using flow cytometry. Previously, increases in the side scatter intensity (SSC) and decreases in forward scatter intensity (FSC) were shown to correlate with changes in the refractive index of cells containing TiO_2_ NPs [58]. Our results reveal shifts in SSC and FSC proving TiO_2_ NPs uptake by ADSCs. Specifically, we found that cultures treated with higher concentrations of TiO_2_ NPs have a larger increase in SSC as compared to ADSCs exposed to lower amounts of NPs. This trend is expected and related to the larger number of NPs inside the cell that can scatter more of the laser beam. Similarly, the larger increase in SSC intensity for anatase containing cells as compared to those with rutile can be explained by the fact that, unlike rutile NPs that are stored in the few vacuoles, anatase is stored in large number of small vacuoles that are much more uniformly distributed in the cytoplasm and thus increases the scattering. It has been shown that SSC provides information on internal structures and organelles [59], therefore, increase in SCC indicates TiO_2_ NPs uptake rather than adhesion to the cell surface. As FSC measures the amount of the laser beam that passes around the cell it can be correlated with relative size of the cell, therefore, a slight decrease in FSC intensity that we observed indicates that cells are roughly the same in size and hence are not apoptotic [58].

Our assessment of TiO_2_ NPs cytotoxicity through the ROS, LDH, and proliferation assays revealed the dose-dependent toxic effects on ADSCs. No cytotoxic effects were observed in cultures treated with 0.1 mg/mL TiO_2_ NPs regardless of crystal structure. In contrast, higher concentrations (0.4 mg/mL) of both, rutile and anatase TiO_2_ NPs, were sufficient to generate oxidative stress and cause LDH release—two hallmarks of NP cytotoxicity. These observations are in agreement with previously reported findings. For example, Shukla et al. reported increased ROS generation in human epidermal cells exposed to TiO_2_ NPs for 6 h [60], similarly other research groups reported concentration dependent increase in ROS in human bronchial epithelial cells [61], brain neurons [62], and human amnion epithelial cells [46]. It is interesting to note, that on day 3 of culture treatment with 0.4 mg/mL TiO_2_ NPs the proliferation profile of ADSCs was not affected even though we detected significant changes in ROS and LDH. Our data revealed a decrease in the cell numbers only after 4 days of exposure to high TiO_2_ NPs concentration confirming greater sensitivity of ROS and LDH assays as compared to a proliferation test.

Similarly, we observed TiO_2_ NP dose-dependent impairment of ADSC wound healing ability. This important cell function was studied using a migration assay and a three-dimensional collagen gel contraction model. Culturing cells in collagen gels is a standard procedure used to evaluate multiple aspects of wound repair and tissue remodeling. This model is used to reproduce behavior of mesenchymal cells and fibroblasts in a “tissue-like” environment [63, 64]. In the gel, cells attach to the matrix by integrin-mediated mechanism, exert mechanical tension and contract the matrix mimicking the wound repair process [63]. Our findings of decreased migration speed and collagen gel contraction in cultures exposed to high TiO_2_ doses can be correlated and interpreted as a reduction of wound healing rates since these cells are required to move into the wound, deposit collagen, and eventually close the wound by contracting the collagen fibers [65, 66]. Such impairment could contribute to ineffective tissue repair and in turn increase chances of bacterial infection. These findings are in agreement with a previously reported decrease in migration and collagen gel contraction in dermal fibroblasts exposed to TiO_2_ [67]. Other nanoparticulate materials, such as SiO_2_, gold, and carbon nanotubes were also reported to have inhibitory effects on collagen gel contraction and migration of cells [68–71].

The importance of interaction between cells and the ECM for regulating proliferation, survival, migration, and differentiation is well established [72, 73]. It is also known that alteration of ECM composition can have a profound effect on cell behavior, including cell migration. For example, previous studies showed that migration of different cell types is promoted by expression and secretion of collagen type I [74–76]. Therefore, our findings of decreased collagen I production in cultures treated with 0.4 mg/mL TiO_2_ NPs may explain the observed reduction in cell migration speed. Collagens are the most abundant structural components of the ECM that support a vast array of cell and tissue functions, including adhesion, migration, differentiation, morphogenesis, and wound healing [77]. It is interesting to note that moderate increases in fibronectin expression alone (in cultures exposed to 0.1 mg/mL TiO_2_) is not enough to alter cell migration. Alternatively, disturbance in the collagen to fibronectin ratio leads to ECM fibrils enriched with fibronectin that makes it softer. Such change in the ECM’s mechanical properties might be partially responsible for suppression of cell migration and collagen contraction. It is well documented, that cells have difficulty to exert proper adhesion and traction forces on softer ECM fibrils [78, 79]. Similarly, alteration of collagen/fibronectin ratio was previously found in human dermal fibroblasts exposed to gold nanoparticles [6]. Further, the role of ADSCs in wound healing was also studied by Kim et al. [80] where he identified secretion of various growth factors by ADSCs as being the essential event that promotes wound closure and re-epithelialization through a paracrine mechanism. For example, different cues secreted by ADSCs activate dermal fibroblasts and keratinocytes which accelerate wound healing in vivo by stimulating collagen expression and migration of dermal fibroblasts, and also protect dermal fibroblasts from oxidative stress [81, 82]. Therefore, we would like to suggest that changes in ADSCs observed in our study may have more profound effects on the wound healing process in vivo due to the complexity of cell–cell interactions and increased secretory load of ADSCs.

Another essential physiological function of ADSCs is their ability to differentiate into adipose tissue and store energy via accumulation of lipids. Adipocyte differentiation is a complex process that occurs via a chain of transcriptional and post-transcriptional events that coordinate changes in cell morphology, hormone sensitivity and gene expression [83, 84]. In this study, we found a delayed lipid accumulation and adipokine secretion in ADSCs that demonstrate the inhibitory effects of TiO_2_ NPs on cell differentiation. Our findings are in agreement with previous reports (including our own previous data) demonstrating similar inhibitory effects of various nanomaterials on adipogenic conversion of ADSCs and mesenchymal stem cells [71, 85, 86]. A recent study evaluated the cytotoxic effects of TiO_2_ nanorods in mesenchymal stem cells [87], however, no changes in adipogenic differentiation were observed. These results may be explained by the low concentration of TiO_2_ nanorods chosen for the study (10 µg/mL).

Since adiponectin is an important mediator of many physiologically relevant processes that help regulate whole body energy expenditure [88], its reduction in various fat depots could potentially generate profound local and systemic effects. For example, it can modify energy metabolism, induce insulin resistance, or promote cardiovascular disease [23, 89–91]. In addition, adipogenesis of dermal adipocytes occurs following injury where during the proliferative stage of the healing process adiponectin-expressing adipocytes repopulate skin wounds [92]. Therefore, reduced adipogenesis of ADSCs exposed to TiO_2_ NPs may also adversely affect skin wound healing in addition to previously observed alterations in ECM expression, migration, and collagen contraction.

The transition of ADSCs from fibroblast-like state to an adipocyte phenotype is a complex event guided by remodeling of the ECM [93] and as such, we studied two main structural proteins of the ECM—fibronectin and collagen during the adipogenic differentiation process. A hallmark of this transition is the degradation of ECM and moderate decrease in fibronectin and collagen amounts. Even though we only observed small fluctuations in fibronectin and collagen content in the control samples, we observed sufficient increase in the amount of both proteins in cultures differentiated in the presence of TiO_2_ NPs. The overproduction of ECM may explain the delayed differentiation observed in the cultures treated with rutile and anatase. On the other hand, such a change in the collagen/fibronectin ratio during differentiation results in stiffer collagen enriched ECM which has been previously reported to limit adipocyte growth [94]. It is interesting that abnormal collagen deposition is a hallmark of fibrosis development in adipose tissue and is tightly linked to tissue inflammation due to infiltration of immune cells [95]. Moreover, increased ECM deposition combined with its decreased flexibility were recently shown to cause adipocyte metabolic dysfunction and obesogenic adipose tissue remodeling [95, 96].

## Conclusions

The discrepancy between in vitro and in vivo cytotoxicity tests results is a bottleneck in developing efficient screening methods to address the safety of a constantly growing number of ENMs. Thus, evaluation of ENMs cytotoxicity based on delivered doses should help to eliminate this difference and enable efficient and reliable in vitro screening methods. However, before developing a new generation of assays the relevance and sensitivity of these tools needs to be carefully assessed.

Here, we have demonstrated greater sensitivity of assays based on specialized cell functions for assessing damage induced by TiO_2_ NPs exposure in ADSCs as compared to the standard assays (ROS, LDH, and proliferation). We observed significant changes in ECM protein secretion and reduction in angiogenesis of cultures treated with low concentrations of TiO_2_ NPs in contrast to no damage detected by standard assays. Such changes potentially can cause impairment of two most important ADSCs functions and lead to unintentional harm to human health. In addition, we demonstrated that TiO_2_ NPs induce cytotoxicity in ADSCs in a concentration-dependent manner by increasing ROS, extracellular LDH, altering ECM protein secretion, and decreasing wound healing ability and differentiation.

Our approach of addressing ENMs toxicity is based on changes in crucial cellular functions which is more sensitive than the set of standardized assays and depicts functional alterations that may have serious health implications. As different cell types will react differently to ENMs exposure, we envision that the proposed approach will help in hazardous ranking of ENMs and also become an essential tool for the development of novel and safer-by-design ENMs.

## Abbreviations

ADSC: adipose derived stromal cells
TiO_2_: titanium dioxide
NPs: nanoparticles
ENMs: engineered nanomaterials
ENM: engineered nanomaterial
PBS: phosphate buffered saline
FBS: fetal bovine serum
DMEM: Dulbecco’s modified eagle’s medium
IBMX: 3-isobutyl-1-methylxanthine
PS: penicillin–streptomycin
ROS: reactive oxygen species
LDH: lactate dehydrogenase
ECM: extracellular matrix
DLS: dynamic light scattering
TEM: transmission electron microscopy
ISDD: in vitro sedimentation, diffusion and dosimetry
VCM: volumetric centrifugation method
PCV: packed cell volume
SSC: side scatter
FSC: forward scatter
